# Vegetative Propagation of *Dictyota kunthii* (Dictyotales, Phaeophyceae) Through Thallus Fragmentation and Ligulae: Potential Alternatives for Cultivation

**DOI:** 10.3390/plants14213387

**Published:** 2025-11-05

**Authors:** Cristian Bulboa, Loretto Contreras-Porcia, Jean Pierre Remonsellez, Camila Mora, Kathya Gomez, Natalia Godoy, Cristian Agurto, Cristian Rogel

**Affiliations:** 1 Centro de Investigación Marina Quintay (CIMARQ), Facultad de Ciencias de la Vida, Universidad Andres Bello, Quintay 2531015, Chile; 2 Instituto Milenio en Socio-Ecología Costera (SECOS), Santiago 8370251, Chile; 3 Center of Applied Ecology and Sustainability (CAPES), Santiago 8331150, Chile; 4 Departamento de Ciencia y Tecnología de los Alimentos (CyTA), Facultad de Farmacia, Universidad de Concepción, Concepción 4070386, Chile; 5 Grupo Interdisciplinario de Biotecnología Marina (GIBMAR) Centro de Biotecnología, Universidad de Concepción, Concepción 4070386, Chile

**Keywords:** *Dictyota kunthii*, Dictyotales, ligulae, growth, seaweeds, culture

## Abstract

The growing interest in the commercial exploitation of the bioactive components of *Dictyota* species, including *Dictyota kunthii* due to its antifungal activity and use in the development of innovative bioproducts, depends on the availability of biomass. In this context, the cultivation of this species emerges as a promising alternative. This study examined thallus fragmentation and ligulae development as methods to produce *D. kunthii*. Accordingly, thalli were divided into apical, middle, and basal sections to generate the respective tissue fragments, which were cultured under controlled conditions. On the other hand, ligulae development was studied under different conditions of photon flux density (10, 35 and 65 µmol m^−2^s^−1^); temperature (10, 17 °C); photoperiod (8:16, 12:12, 16:08 h [Light:Dark]), and seawater enrichment:Basfoliar^®^, Compo Expert, Krefeld, Germany and von Stosch solutions. The results show that fragmented thalli were non-viable, exhibiting neither wound healing nor regeneration at the cut sites. Furthermore, no buds or new branches were formed. In contrast, ligulae developed under all tested conditions, with nutrients, light, temperature, and photon flux enhancing apical cell formation and branching. We conclude that ligulae can effectively be used as propagules to cultivate fast-growing, branched *D. kunthii* plantlets. Accordingly, we recommend using a suspended culture system at 17 °C with a 12:12 (Light:Dark) photoperiod and 65 µmol m^−2^ s^−1^ light intensity, as well as adding nutrients (Basfoliar^®^ at 0.1 mL L^−1^). Under these conditions, growth rates equal to or exceeding 10% d^−1^ can be achieved, supporting the feasibility of scaling up to larger volumes for biomass production.

## 1. Introduction

*Dictyota kunthii* is a brown seaweed that grows in both shallow subtidal and intertidal pools across temperate to subtropical zones [1]. It is commonly found in Chile, specifically from Arica to Chiloe, where it forms small, scattered patches [2]. Species of this genus are widely studied due to its active components, including groups such as terpenes, diterpenes, polyunsaturated fatty acids, phenolic compounds, and polysaccharides, which exhibit well-characterized bioactivity, e.g., [3,4,5,6,7,8].

Particularly, the antifungal activity of *Dictyota* has been addressed by different studies. For example, the methanolic extracts of *D. linearis* and *D. dichotoma* [9] showed high activity against the fungi *Aspergillus niger*, *Aspergillus flavus*, *Candida utilis*, *Fusarium solani*, and *Penicillium* sp. These findings have also been evidenced in both *Dictyota* species against phytopathogens and human-pathogenic fungi [10]. Furthermore, nanoparticles obtained at the laboratory scale from *D. bartayresiana* extracts (from natural grasslands) showed antifungal activity against *Humiclo insulans* and *Fusarium dimerum* [11]. Additionally, *D. menstrualis* bioautography assays indicated antifungal and antioxidant potential of the organic extract and fractions against *Cladosporium sphaerospermum* [12].

Regarding the antifungal properties of *D. kunthii* algae extracts, three patents have demonstrated its activity, and it has been utilized in innovative bioproducts such as bioplastics, bioactive papers, bioactive sleeves, and formulations for protecting export fruits [13,14,15]. These products have reduced fruit loss due to oxidation and infection in both laboratory and industrial trials. Thus, *D. kunthii* extracts inhibited the germination of fungi such as *Alternaria alternata*, *Penicillium* spp., and *Botrytis cinerea*, halting their growth for over 90 days. For instance, foam sleeves reduce *B. cinerea* infection in apples by 53%, and bioactive papers decrease losses due to infection by *B. cinerea* and *Penicillium* spp. by 50 to 70% [13,14]. Additionally, studies have shown the effectiveness of *D. kunthii* extracts against forest pests such as the eucalyptus weevil and the pine bark beetle, with a repellency of 87% and lethality of 67% for *H. ligniperda* and 100% lethality for *G. platensis* [15]. The high bioactivity, chemical complexity, and compounds of *Dictyota* species have triggered growing market interest, resulting in future potential demand for biomass. Therefore, understanding the lifecycle, reproduction, and preservation of natural *Dictyota* populations is crucial for formulating strategies for domestication and controlled propagation, e.g., [16].

Species of the order Dictyotales have a diplohaplontic lifecycle with isomorphic, sporophytic, and gametophytic thalli [17]. The lifecycle presents different propagation pathways, which consider both sexual and asexual strategies. Gametophytic (haploid) thalli are dioecious and produce sori of oogonia and antheridia, which release gametes into the environment where they fuse to form a zygote, giving rise to sporophytic (diploid) thalli. These, through meiosis, produce spores in tetrads along the thallus, which after germination reestablish the haploid phase. Additional important asexual pathways contributing to the maintenance of natural populations include thallus fragmentation into re-attachable units [18] and the presence of perennial basal or prostrate structures capable of generating new thalli [19]. In the case of *D. kunthii*, the presence of ligulae (spatulate protrusions on the thallus surface) provides an additional method of asexual reproduction. Detached ligules are thought to regenerate entire thalli [2,20], although this has not yet been demonstrated. For many algae species, the alternation of generations is balanced, with sexual and asexual reproduction occurring at similar intensities [21]. However, *Dictyota* species are an exception: in several species within the Dictyotales order, the presence of fertile gametophytes has been reported as rare or completely absent [22,23,24]. This is consistent with the description by [25] of *D. kunthii* along the central Chilean coast, where only sporophytic and infertile thalli were observed throughout an annual cycle. This implies that, in many cases, the persistence of *Dictyota* populations depends on asexual reproduction, where reproduction via spores would be one of the most important propagation strategies in alternation with thallus fragmentation and subsequent reattachment [26].

Optimal macroalgal cultivation depends on the balance of nutrient availability and physical factors, which together regulate growth, morphogenesis, and reproduction under confined conditions. In seaweed culture systems, parameters such as light intensity, photoperiod, temperature, and seawater nutrient composition strongly influence metabolic activity and cellular differentiation [27,28,29,30,31]. Adequate nutrient supply, particularly nitrogen, phosphorus, and trace elements, supports protein synthesis, pigment formation, and tissue regeneration, while deficiencies can rapidly lead to growth inhibition or thallus decay [32,33]. Moreover, hydrodynamic conditions and aeration play critical roles by enhancing CO_2_ diffusion and nutrient uptake, thereby sustaining photosynthetic efficiency and structural integrity [28]. The evaluation of these abiotic variables under controlled laboratory conditions provides a basis for identifying optimal combinations that promote sustained growth. Thus, understanding such physiological responses is essential for defining suitable cultivation strategies for *Dictyota kunthii*.

Both spores and fragmentation could serve as potential methods for the domestication and cultivation of certain species, thereby bypassing the more complex process of sexual reproduction. Indeed, sexual reproduction requires finding male and female individuals and involves several complicated steps such as settlement, germination and growth of the new individuals, often requiring controlled laboratory conditions [21]. Propagation via spores or fragmentation remains untested in this genus, and spore-based approaches are notably delicate and labor-intensive, especially for generating biomass at scales suitable for bioactive compound extraction. Although there are no commercial cultures of *Dictyota* spp., there are instances where both spores [20,25,34] and thallus fragments [16] have been successfully cultured in the laboratory [35]. These studies demonstrate the ability of *Dictyota* species to grow and reproduce under confined conditions, albeit limited to the laboratory scale. For *D. dichotoma* [34], the plants remained healthy and retained their typical morphology, and many clones could be obtained by asexual reproduction through spore culturing. Similarly, fragmentation appears to be a powerful strategy for productive *Dictyota* cultivation. Research by [36] demonstrated that *Dictyota dichotoma* thalli can be broken into different pieces, which regenerate and regrow after wounding. In this context, the literature suggests that *D. kunthii* culture could be initiated using fragments or ligulae through standard algal culture methods. However, further research is needed to determine which strategy can be scaled up for biomass production. Accordingly, this study aimed to evaluate the vegetative propagation of *D. kunthii* via fragmentation and the growth of ligulae under controlled laboratory conditions. The results are expected to contribute significantly to enabling large-scale biomass production.

## 2. Results

### 2.1. Thalli Fragmentation and Ligulae

**Figure 1:**
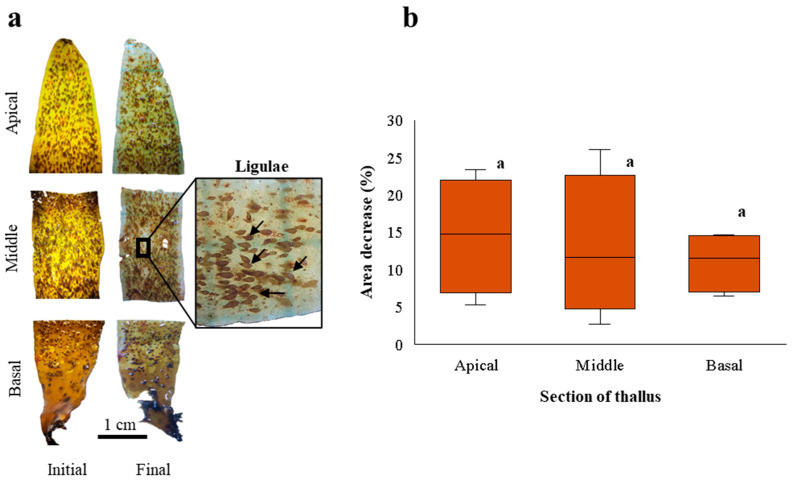
(**a**). Apical, middle, and basal fragments of *D. kunthii* thalli at the beginning (initial) and end (final) of the experimental period. The inset shows abundant ligulae (indicated by black arrows) on the thallus. (**b**). Area decrease (%) of apical, middle, and basal fragments of *D. kunthii* thalli cultivated under controlled laboratory conditions for 21 days. Letters above the bars indicate statistical differences (*p* < 0.05). Values shown as the median ± d 90% quartiles, n =  4 per treatment.

After 21 days of culture, the fragments of the apical, middle, and basal sections of *D. kunthii* thalli showed losses of coloration, turgor, and, in some cases, tissue loss, demonstrating a deterioration with respect to the initial condition (Figure 1a). No evidence of regeneration or bud formation was observed at the cut sites or elsewhere on the thallus. However, for all fragments, surface ligulae were abundant and exhibited natural coloration without tissue loss or evident deterioration (Figure 1a). GR was negative for the different sections of the thallus: −1.2 ± 0.6; −1.2 ± 1; and −0.8 ± 0.3% d^−1^ for the apical, middle, and basal fragments, respectively. A decrease in area was also recorded for the apical (15 ± 7.8%), middle (13 ± 9.7%), and basal (11 ± 4.1%) fragments (Figure 1b), although without statistical differences (F = 0.218; *p* > 0.05).

The ligulae developed and grew under all treatments, in some cases forming highly branched thalli by the end of the experimental period. The formation of reproductive structures in the ligulae (tetraspores) was not observed for any treatment.

### 2.2. Effect of Photon Lux Density (PFD)

**Figure 2:**
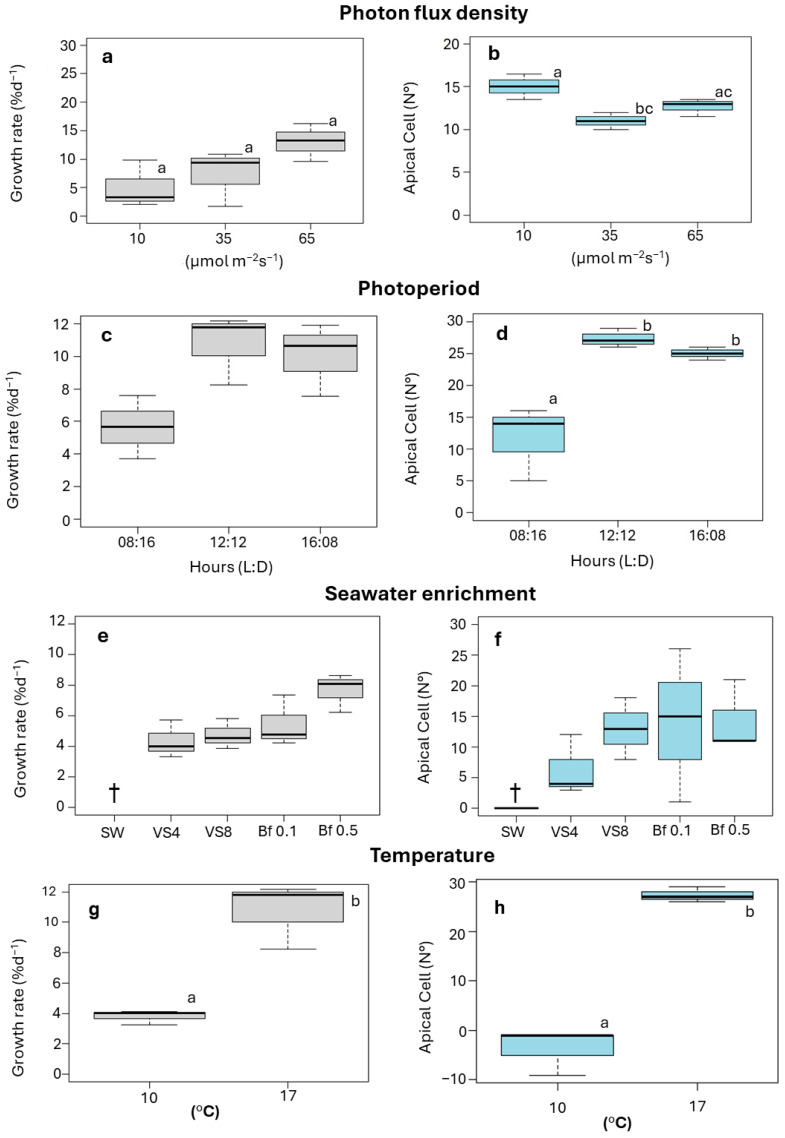
Growth rate (GR, % d^−1^) and number of apical cells of ligulae cultivated under selected abiotic factors. (**a**,**b**). photon flux density; 10, 35, and 65 µmol m^−2^s^−1^. (**c**,**d**). photoperiod; 08:16, 12:12, and 16:08 h (Light:Dark). (**e**,**f**). seawater enrichment; SW (seawater), VS4 (von Stosch 4 mL L^−1^), VS8 (von Stoch 8 mL L^−1^), Bf 0.1 (Basfoliar^®^ 0.1 mL L^−1^), and Bf 0.5 (Basfoliar^®^ 0.5 mL L^−1^). (**g**,**h**). temperature, 10 and 17 °C. Letters above the bars indicate statistical differences (*p* < 0.05). Values shown as the median ± 90% quartiles, n =  3 per treatment. †: total mortality of ligulae biomass.

**Figure 3:**
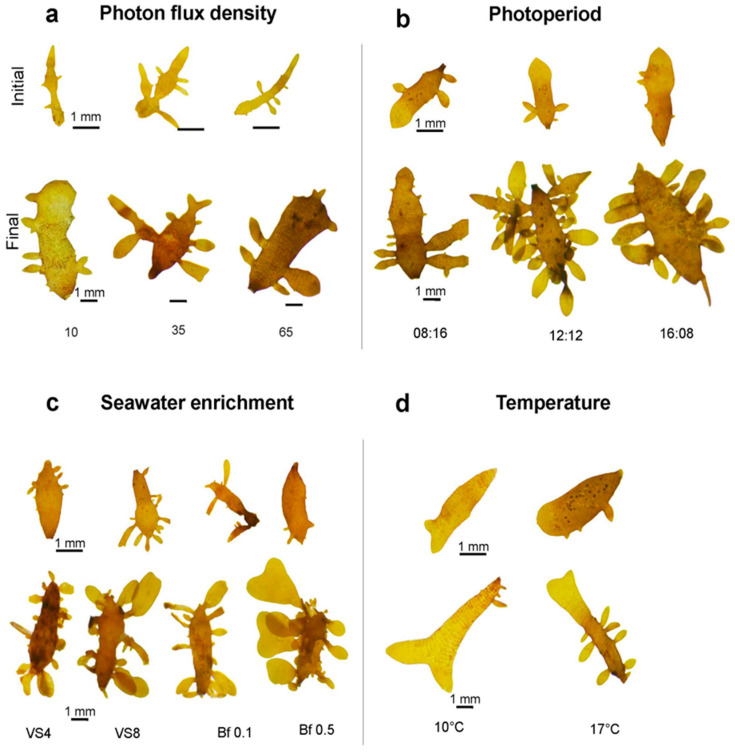
Representative images of the external morphological changes in ligulae cultivated for 21 days under different abiotic factors: (**a**). photon flux density; 10, 35, and 65 µmol m^−2^s^−1^. (**b**). photoperiod; 08:16, 12:12, and 16:08 h (Light:Dark). (**c**). seawater enrichment; VS4 (von Stosch 4 mL L^−1^), VS8 (von Stoch 8 mL L^−1^), Bf 0.1 (Basfoliar^®^ 0.1 mL L^−1^), and Bf 0.5 (Basfoliar^®^ 0.5 mL L^−1^). (**d**). temperature, 10 and 17 °C.

An increase in ligulae growth was observed with increasing PFD (Figure 2a). Growth was lowest at 10 µmol m^−2^ s−^1^, with a GR of 2 ± 0.2% d^−1^. The highest GR was achieved at 65 µmol m^−2^ s^−1^, with a value of 15 ± 0.2% d^−1^ (Figure 2a). However, these differences were not significant (F = 2.869; *p* > 0.05). The number of apical cells produced by the ligulae was significantly influenced by PFD (F = 2.659; *p* < 0.05), the highest apical cell count (15 ± 4) was observed at 10 µmol m^−2^ s^−1^, while the lowest (7 ± 2) occurred at 35 µmol m^−2^ s^−1^ (Figure 2b). At the end of the culture period under 10 µmol m^−2^ s^−1^, the ligulae exhibited a loss of coloration and turgor (Figure 3a).

### 2.3. Effect of Photoperiod

Ligulae cultivated under different photoperiod treatments showed a similar variation for both GR and production of apical cells, with the lowest values of both variables recorded at 08:16 h (Light:Dark) (Figure 2c,d). The highest GR was obtained with increasing light hours, reaching a GR of 12 ± 0.1% d^−1^ at 12:12 h (Light:Dark) (Figure 2c). However, no statistical differences between treatments were recorded (F = 5.0862; *p* > 0.05). The different photoperiod treatments did show significant differences in the number of apical cells produced (F = 17.07; *p* < 0.05). The highest number (27 ± 1.5 cells) was reached in the 12:12 h (Light:Dark) treatment, while the lowest value (12 ± 6.4 cells) was observed in the 8:16 h (Light:Dark) treatment (Figure 2d). At the end of the experimental period, a higher number of apical cells were produced in the treatments at 12:12 h (Light:Dark), resulting in ligulae with abundant branching (Figure 3b).

### 2.4. Effect of Seawater Enrichment

The ligulae exhibited greater growth and development when cultured in enriched seawater (Figure 2e,f). Conversely, ligulae cultured in seawater without nutrient supplementation exhibited progressive decay, ultimately resulting in total mortality by the end of the experimental period (Figure 2e,f). The GR varied from 4 ± 0.03 to 8 ± 0.04% d^−1^ for the von Stosch (4 mL L^−1^) and Basfoliar^®^ (0.5 mL L^−1^) treatments, respectively (Figure 2e). Although the growth was higher with Basfoliar^®^ enrichment, no significant differences were observed between treatments (F = 3.755; *p* > 0.05).

The formation of apical cells was favored in the treatments with Basfoliar^®^ (Figure 2f), reaching a maximum value of 14 ± 12 cells with Basfoliar^®^ 0.1 mL L^−1^. The von Stosch treatment (4 mL L^−1^) recorded the lowest production of apical cells (5 ± 4 apical cells; Figure 2f). However, these differences were not statistically significant (F = 0.733; *p* > 0.05). At the end of the experimental period, the ligulae exhibited abundant dichotomous branching (Figure 3c).

### 2.5. Effects of Temperature

A GR of 11 ± 0.1% d^−1^ was reached at 17 °C, but at 10 °C was significantly lower (4 ± 0.01% d^−1^) (T-Student, *p* = 0.0038) (Figure 2g). The production of apical cells varied significantly between treatments at 17 °C and 10 °C (T-Student, *p* = 0.0265) (Figure 2h). The 17 °C treatment was more favorable, resulting in 27 ± 2 apical cells, while the 10 °C treatment caused a loss of these cells. Despite the temperature differences, the ligulae remained healthy in both treatments, showing typical coloration and numerous small branches (Figure 3d).

## 3. Discussion

*Dictyota* spp. exhibit substantial phytochemical diversity, containing many biocomponents with applications in high-value sectors, such as pharmaceuticals, nutraceuticals, and cosmetics [37,38]. Additionally, *D. kunthii* has recently been reported to exhibit antifungal properties and potential for application in forest pest control [13,14,15]. The extraction of these biocomponents requires high-quality biomass; thus, cultivation is a proposed alternative to the uncertain supply of natural populations. Nevertheless, the methods and conditions that facilitate *Dictyota* productive cultivation have been scarcely investigated [16], and, due to the characteristics of the *Dictyota* thallus, few studies suggest that vegetative fragmentation could be a viable alternative for suspended culturing [5,16,35], with no commercial cultures reported in the literature. The present results indicate that cultivation of *D. kunthii* via thallus fragments is not advisable. However, the presence of ligulae on the thallus surface appears to represent a viable alternative for propagation.

Unlike previous reports on other *Dictyota* species [18,29], the results presented here indicate that *Dictyota kunthii* thalli do not produce viable fragments. Tanaka et al. [36] showed that various parts of the *D. dichotoma* thallus can recover after fragmentation, with the ability to maintain apical growth, regenerate branches, or form rhizoids, actively responding to wound stress. These characteristics promote the formation of thallus fragments, which have been identified as one of the main strategies for propagating and maintaining natural populations in several *Dictyota* species [16,18,39]. However, for *D. kunthii*, the cut sections of the thallus did not exhibit wound healing, thallus regeneration, buds, or new branches, instead evidencing turgor loss and discoloration. In this regard, Malbrán and Hoffmann [25] observed that asexual strategies, such as spores and perennial basal disks, are the most reliable mechanisms for maintaining *D. kunthii* populations in central Chile, over thallus fragmentation.

Additionally, ligulae have been proposed as an additional strategy for propagating *D. kunthii* [20,25], although this has not yet been documented in situ, as the reattachment and subsequent development of ligulae in adult thalli remain unconfirmed. However, our results add evidence supporting this idea as; (i) ligulae persisted despite the disintegration of fragmented thalli, (ii) grew actively in different culture conditions, and (iii) produced branched thalli like the parents. Taken together, these findings indicate that ligulae, as propagules, can be expected to prevail over thallus fragmentation and develop new thalli, although ligulae reattachment mechanisms must be clarified.

The present study showed that ligulae growth is actively influenced by abiotic factors such as PFD, temperature (17 °C), and photoperiod, which aligns with descriptions by Hoffmann [27] and Hoffmann & Malbrán [20]. However, the current findings further indicate that development can also be stimulated by nutrient-enriched cultures. This is consistent with reports for several *Dictyota* species that can rapidly and efficiently uptake available nutrients [37]. In the present study, ligulae grew in all experimental treatments, actively forming apical cells. By contrast, the seawater control treatment led to complete mortality of the ligulae. Consistent with these findings, the nutrient addition to accelerating biomass production is common and necessary in confined cultures without permanent seawater flow [32,33], especially in seedling or propagule production [40]. Nonetheless, nutrients can be very costly to apply. Therefore, the use of small amounts of fertilizer solutions or biostimulants, such as Basfoliar^®^, has become a productive option, especially in closed cultures. Both are increasingly being used in species of commercial interest, such as *Macrocystis pyrifera* and *Gracilaria chilensis*, being cost-effective and having excellent production results [30,41,42]. These outcomes are particularly notable when compared to traditional solutions for seawater enrichment in algal cultures, such as use of the von Stosch medium [30]. The present results showed that the addition of enrichment solutions (e.g., Basfoliar^®^, from 0.1 mL L^−1^) not only increased growth, but induced secondary development of the ligulae, promoting the formation of plantlets with abundant branching, which facilitates later cultivation stages. In this regard, we have recently observed that unbranched ligulae are unable to float and precipitate. This makes suspended culture in hatchery tanks (>300 L) and contributes to increased mortality (unpublished data).

Hoffmann [27] and Hoffmann and Malbrán [20] demonstrated a close association between temperature, photoperiod, and PFD with fertile ligulae (i.e., tetraspore formation). In contrast, the present study did not observe tetraspores on the ligulae in any of the experimental treatments, even under short-day conditions, which [27] mentioned as a trigger for tetrasporogenesis. Furthermore, in vitro tetraspore production has been documented to coincide with low ligulae development [20], suggesting that both processes are differentially stimulated by abiotic factors. This is advantageous for scaling ligulae-based cultivation since investing energy in growth and avoiding senescence after reproduction is desirable [43]. The absence of reproductive structures could be due to the permanent aeration used in this study, which would favor thallus growth. In fact, moderate water motion or aeration enhances boundary-layer thinning, improving the delivery of dissolved inorganic carbon and nutrients to the algal surface, which in turn favors rapid thallus growth [44]. In this sense, prior research proposes that hydrodynamic conditions may favor growth by improving CO_2_ availability and nutrient uptake [28,29,32]. Therefore, the permanent aeration in the present study may have created conditions that favored sustained growth and branching at the expense of tetrasporogenesis. Nevertheless, this hypothesis warrants further investigation, particularly through experiments that compare static versus aerated conditions to determine the specific hydrodynamic thresholds influencing reproductive induction in *D. kunthii*.

## 4. Materials and Methods

Thalli of *D. kunthii* were collected manually, ensuring complete removal of each individual. from Algarrobo Bay (33°21′53.79″ S 71°40′46.30″ W) Valparaíso Region, central Chile, during autumn 2023. The site was chosen based on the natural abundance of thalli in the mid- to low-intertidal zones along the central Chilean coast [25]. The thalli were transported wet to the laboratory in hermetic bags kept at 10 °C. Once in the laboratory, we selected healthy thalli that displayed adequate coloration and free of epibionts visible to the naked eye. Additionally, the basal attachment disk was excised from each alga, as these parts typically contain sand and epibiotic organisms. Subsequently, the biomass was thoroughly rinsed with freshwater to remove sediment, associated macroalgae, and invertebrates, followed by rinsing with filtered seawater (1 µm) and a 9% iodine solution. Subsequently, 30 thalli were selected and maintained in an aquarium with 10 L of filtered (1 µm) seawater (32 PSU) enriched with the von Stosch [45] seawater medium solution (8 mL L^−1^), for 20 days to acclimatize the thalli to laboratory conditions and ensure that they remain healthy for the start of experiments. The aquariums were kept at 17 ± 1 °C, with bubbling aeration, a 12:12 h photoperiod (Light:Dark), a photon flux density (PFD) of 40 µmol m^−2^ s^−1^, and weekly seawater renewal. All experiments detailed below were conducted using this biological material.

### 4.1. Thallus Fragmentation: Growth and Regeneration

**Figure 4:**
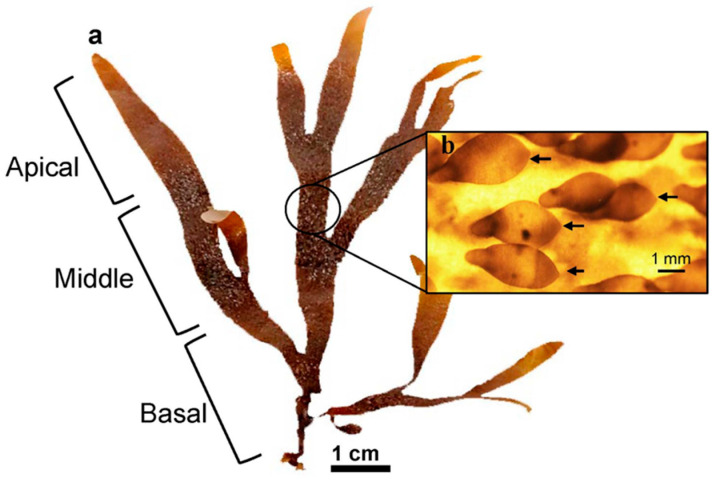
(**a**). Thallus of *D. kunthii* indicating the apical, middle, and basal sections used to obtain fragments. (**b**). Detail of unbranched ligulae (black arrows) with a single apical cell.

Thalli were cut into three sections along the algal body, producing apical fragments (up to 3 cm below the apex), basal fragments (3 cm from the base), and middle fragments (Figure 4a). Four 500 mL Erlenmeyer flasks were used for each type of fragment, with three pieces arranged in each flask independently (0.1 g L^−1^). Fragments were maintained for 21 days under the specified conditions, with weekly medium renewal. All fragments were observed using a Motic SMZ-140 stereo microscope, and the photographs were processed using the ImageJ software v1.53. The area of each fragment was measured at the beginning and end of the culture period. The growth rate (GR) was calculated using the formula proposed by [46]: GR (%d^−1^) = [[(Ai/Af)1/t − 1] × 100], where Ai = initial area and Af = final area after t days. Changes in area during the experimental period were expressed as a percentage relative to the initial area. Thallus regeneration was also assessed by observing the fragments under a Motic SMZ-140 stereoscopic microscope (Motic Instruments, Speed Fair Co., Ltd., Universal City, TX, USA). The number of fragments that developed shoots and the total number of shoots per treatment (i.e., apical, middle, and basal fragments) were recorded. Regeneration was expressed as a percentage (%) of the total number of fragments that produced shoots.

### 4.2. Ligulae: Growth and Development

Thalli with abundant ligulae were selected, ensuring that only small ligulae (i.e., 1–4 mm in length) without visible branching, containing only one or a few apical cells were chosen (Figure 4b). The ligulae were separated from the thalli using a scalpel by gently scraping the surface. Then, the mass obtained was washed with abundant filtered seawater (1 µm). Prior to the experiments, ligulae were incubated in a 4 L seawater container under the previously described conditions for 10 days to allow the identification and removal of mechanically damaged individuals. A single-factor experiment (Table 1) was performed to evaluate the influence of PFD, photoperiod, seawater enrichment (von Stoch solution and Basfoliar^®^, according to [30]) (Table 2), and temperature on the growth and the number of the apical cells for *D. kunthii* ligulae. Each experiment was carried out for 21 days (We defined a cultivation period of less than one month, as we want to find efficient cultivation conditions to accelerate the seedling or plantlet production stages, since extended periods of cultivation under controlled conditions tend to be costly). For this purpose, 500 mL Erlenmeyer flasks were filled with filtered seawater (1 µm) and maintained under the conditions detailed in Table 1, in a suspended-culture system. For each treatment, three Erlenmeyer were used, into which 0.1 g of ligulae (fresh weight ~ 70–100 ligulae) were added. Seawater and the culture medium were renewed weekly.

At the beginning and end of each experiment, the wet weight of the ligulae was measured using an analytical balance (Boeco Bas 31 plus), and the GR (% d^−1^) was calculated using the previously described formula. The number of initial and final apical cells after 21 days of culture was determined by observing and counting 15 randomly selected ligulae in each experiment, using a stereoscopic microscope (Motic SMZ-140). Additionally, the surface of the ligules was examined during each measurement to detect spore formation (tetrasporangia).

### 4.3. Data Analysis

Normality was assessed with a Shapiro-Wilk test and homoscedasticity with Bartlett’s test. To determine differences in growth and survival among thalli sections (i.e., apical, middle, and basal) an ANOVA test (one-way) was conducted, followed by the Tukey test when necessary. On the other hand, a one-way ANOVA was performed to evaluate the effects of PFD, photoperiod, and the seawater-enrichment solution on the variables of GR and number of apical cells. Post hoc Tukey tests were performed to assess treatments showing significant differences. For temperature comparisons between treatments were performed using Student’s *t*-test.

## 5. Conclusions

Algae cultivation is usually carried out in multi-step systems considering production from seed to harvest [40]. The present study demonstrated that ligulae can be used as propagules for the cultivation of *D. kunthii* since they grow actively under different conditions, rapidly modifying in shape and forming plantlets with abundant branching (21 days). This is an advantage, as extensive plantlet production stages can be very costly, making the entire process inefficient. To accelerate the ligulae-to-propagule transition and to ultimately obtain a plantlet-like adult thallus able to develop and produce biomass, we recommend a suspended culture system (permanent aeration) with a temperature of 17 °C, a 12:12 Light:Dark, a PFD of 65 µmol m^−2^s^−1^, and nutrient addition (Basfoliar^®^ (0.1 mL L^−1^)). According to this study, growth rates ≥ 10% d^−1^ could be achieved under these conditions, meaning that ligulae could double in size in a few days, facilitating potential scaling up to larger volumes for producing biomass.

## Figures and Tables

**Table 1:** Summary of factors and levels used to evaluate the growth of *D. kunthii* ligulae and apical cell formation under controlled conditions. Aeration was added in all treatments.

Experimental Factor	Levels	Culture Conditions (Remain Constant)
Photon flux density (PFD) (µmol m^−2^s^−1^)	10, 35, 65	12:12 h (Light:Dark) von Stosch (8mL L^−1^) 17 °C
Photoperiod (Light:Dark)	8:16, 12:12, 16:08	35 (µmol m^−2^ s^−1^) von Stosch (8mL L^−1^) 17 °C
Seawater enrichment solution (mL L^−1^)	Basfoliar^®^ 0.1 Basfoliar^®^ 0.5 von Stosch 8 von Stosch 4 Seawater (1 µm)	35 (µmol m^−2^ s^−1^) 12:12 h (Light:Dark) 17 °C
Temperature (°C)	10 17	35 (µmol m^−2^s^−1^) von Stosch (8mL L^−1^) 12:12 h (Light:Dark)

**Table 2:** Composition of the culture media used. Modified from [30].

Von Stosch	Basfoliar ^®^
NaNO_3_ (0.0875 g N 100 mL^−1^)	Total nitrogen (4.17 g 100 mL^−1^)
Na_2_HPO_4_ · 12 H_2_O (0.01164 g P 100 mL^−1^)	P_2_O_5_ (37.5 g 100 mL^−1^) (16.4 g P 100 mL^−1^)
Vitamin B_1_	K_2_O (25 g 100 mL^−1^)
Vitamin B_12_	B (0.014 g 100 mL^−1^)
Vitamin B_7_-B_8_	Cu [Cu(II)-EDTA] (0.028 g 100 mL^−1^)
FeSO_4_ · 7H_2_O (0.00869 g 100 mL^−1^)	Fe [Fe(II)-EDTA] (0.07 g 100 mL^−1^)
MnCl_2_ · 4H_2_O (0.00124 g 100 mL^−1^)	Mn [Mn(II)-EDTA] (0.014 g mL^−1^)
EDTA (0.0465 g 100 mL^−1^)	Mo (0.0012 g 100 mL^−1^)
	Zinc (0.014 g 100 mL^−1^)
Mass ratio N/P in culture medium Von Stosch 5:1 Basfoliar ^®^ 1:4	

## Data Availability

Data are contained within the article.
